# Thyroid Autoantibodies in Pregnancy: Their Role, Regulation and Clinical Relevance

**DOI:** 10.1155/2013/182472

**Published:** 2013-04-18

**Authors:** Francis S. Balucan, Syed A. Morshed, Terry F. Davies

**Affiliations:** Thyroid Research Unit, Mount Sinai School of Medicine and James J. Peters VA Medical Center, 130 West Kingsbridge Road, Bronx, New York, NY 10468, USA

## Abstract

Autoantibodies to thyroglobulin and thyroid peroxidase are common in the euthyroid population and are considered secondary responses and indicative of thyroid inflammation. By contrast, autoantibodies to the TSH receptor are unique to patients with Graves' disease and to some patients with Hashimoto's thyroiditis. Both types of thyroid antibodies are useful clinical markers of autoimmune thyroid disease and are profoundly influenced by the immune suppression of pregnancy and the resulting loss of such suppression in the postpartum period. Here, we review these three types of thyroid antibodies and their antigens and how they relate to pregnancy itself, obstetric and neonatal outcomes, and the postpartum.

## 1. Introduction: Pregnancy and Tolerance

Immune tolerance in pregnancy is the absence of a maternal immune response against the fetus and placenta, leading to unusually successful “allografts” as the fetus is genetically different from the mother. This adaptive phenomenon involves all aspects of the immune response [1], but in this review, we will concentrate on those aspects directly and indirectly related to thyroid autoantibodies.

## 2. Thyroid Autoantibodies to Thyroglobulin and Thyroid Peroxidase

### 2.1. Discovery

In 1925, Hektoen and Schulhof [2, 3] in their animal studies with thyroglobulin (Tg) precipitins proposed that Tg provoked an immune response. Later, it was noticed that *γ*-globulins were increased in patients with Hashimoto's thyroiditis, which were then theorized to reflect autoantibodies against Tg [4]. With this information and animal studies from Witebsky et al. [5] that thyroiditis could be induced by Tg in rabbits, Campbell et al. (1956) demonstrated the possible role of antibodies to Tg in Hashimoto's thyroiditis [6]. The subsequent development of clinical assays for both Tg and Tg-Ab [7] improved our information about the pathology of the disease, and it is clear that Tg is a major autoantigen in both Hashimoto's and Graves' diseases and its immunogenicity varies with its degree of iodination [8]. Whole-genome linkage studies have shown that the chromosome 8q24 locus [9], which contains the Tg gene, is linked to the development of autoimmune thyroiditis [10] suggesting that polymorphisms in the gene are an important mechanism for provoking the immune response [11].

Thyroid peroxidase (TPO) was first discovered as a thyroidal microsomal autoantigen by Belyavin and Trotter in 1957 [12]. It took 20 years for this autoantigen to be characterized as TPO by Portmann et al. [13]. TPO-Abs are present in much higher titers than Tg-Ab since, unlike Tg the TPO enzyme is membrane bound and not secreted into the circulation and so does not cause TPO-Ab to be removed or blocked from being measured. 

### 2.2. Structure of the Thyroid Antigens

**Figure 1:**
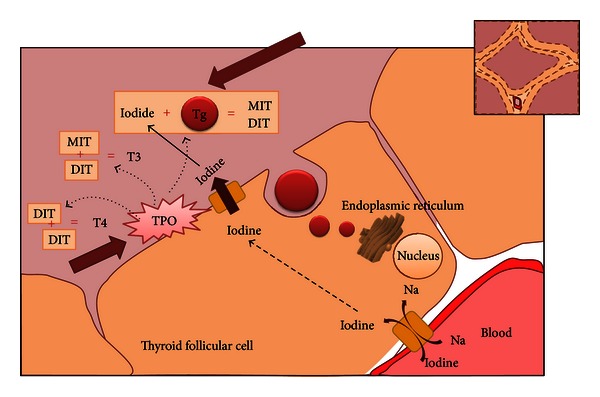
Thyroglobulin and thyroid peroxidase in thyroid hormone biosynthesis. The essentiality of thyroglobulin and thyroid peroxidase is shown in this simplified diagram of thyroid hormone biosynthesis. Thyroglobulin acts as the skeleton in thyroid hormone synthesis. It is synthesized and glycosylated in the rough endoplasmic reticulum, and is exocytosed through the apical membrane. Thyroid peroxidase acts in three parts (a) it oxidizes iodine, (b) organifies iodide to the tyrosine residues to the thyroglobulin forming monoiodotyrosine and di-iodotyrosine (c) Couples monoiodotyrosine and di-iodotyrosine to either T4 or T3 (TPO: thyroid peroxidase, Tg: thyroglobulin, MIT: monoiodotyrosine, DIT: di-iodotyrosine, T4: thyroxine, and T3: triiodothyronine).

Both Tg and TPO are essential in the synthesis and secretion of thyroid hormones (Figure 1). The thyroglobulin gene on 8q24.2 codes for a large dimeric glycoprotein of 330 kd for each dimer [14, 15]. Tyrosine residues of Tg homodimers are iodinated in the apical border of the thyroid cell to form monoiodotyrosine (MIT) and di-iodotyrosine (DIT) [9]. The importance of Tg in thyroid hormone synthesis is emphasized by mutations in the Tg gene, which may lead to various degrees of congenital hypothyroidism [16]. Thyrotropin (TSH) stimulates the expression of Tg via the TSH receptor, but its expression is not TSH dependent, since it can be maintained by IGF-1 and other growth factors [17]. 

The TPO gene on chromosome 2p25 is also regulated by TSH, but in this case it is much more transcriptionally TSH dependent than Tg with expression lost in the absence of TSH. TPO is a membrane associated protein of approximately 107 kDa and expressed mostly on the apical surface of the thyrocyte. It is thyroid peroxidase which catalyzes the coupling of two molecules of DIT or one of DIT and MIT for the formation of T4 and T3, which is then stored in the colloid as part of the Tg molecule [14].

### 2.3. Characteristics of TPO-Ab and Tg-Ab

Antibodies are B-cell produced Y-shaped proteins that are essential for the identification and neutralization of bacteria and viruses [19]. Autoantibodies which are pathologic to *self* molecules have been the *sine qua non* of autoimmune disease, including the autoimmune thyroiditides [20]. In addition, natural autoantibodies present in all of us and which are present without exposure to a foreign antigen [21] may also have a *functional role* in the immune response [22]. The autoimmune responses to Tg and TPO are polyclonal and the result, therefore, of multiple gene involvement. The autoantibodies to Tg and TPO are predominantly of the immunoglobulin (Ig) G class [18, 23] although lower levels of IgA class Tg-Ab and TPO-Ab can also be found in patients with AITD [24, 25] and they can be found in all IgG subclasses [26]. Using a variety of monoclonal TPO-Ab Fab preparations, it has been noted that serum TPO-Abs interact with a restricted region on TPO, termed the *immunodominant region*, recognized by >80% of individuals [27, 28]. Although TPO-Ab and Tg-Abs are both markers of thyroid autoimmunity, it has been argued that TPO-Ab is more relevant in the prediction of thyroid dysfunction (Table 1) [18]. Whether TPO is a more important thyroid autoantigen than Tg is doubtful, since its gene is not associated with AITD [29], although there is evidence for inheritance of an IgG subclass pattern of expression [30]. Since both TPO and Tg can initiate thyroiditis in animal models, this suggests that both are potent autoantigens [2, 12].

## 3. Thyroid Autoantibodies in the Normal Population

### 3.1. Epidemiology

In the original Whickham study in the United Kingdom, performed in the 1970s, it was estimated that the prevalence of Tg-Ab was 2% in the sample of 2799 people, while TPO-Abs were present in 6.8% of the sample [31]. The frequency increased with age in females, but not in males and only 2.2% of males compared with 8% of females had TPO-Ab. The National Health and Nutrition Examination Survey (NHANES) data from the USA reviewed in 2002, examining 17,353 people, showed TPO-Ab in 13% and Tg-Ab in 11.5% of the population using more sensitive assays. Similar to the findings in the Whickham study followup, there was an increase in thyroid autoantibodies with age in females. NHANES also showed that the prevalence of TPO-Ab and Tg-Ab was higher in Caucasians, as compared to African Americans [32]. 

### 3.2. Regulation of the B-Cell: The Source of Autoantibodies

B-cells are lymphocytes that play a major role in the humoral immune response including immunoglobulin (Ig) secretion and antigen presentation. They are usually divided into two lineages. The first is the B1 lineage which produces polyreactive antibodies of the IgM class, often referred to as natural antibodies that do not undergo hypermutation of their variable genes. The second is the B2 lineage which is capable of generating a variety of antibodies of different Ig classes and subclasses [33]. The genetic recombination of V, D, and J region segments of the immunoglobulin heavy chains, followed by similar recombination in the light chain of the V and J segments happens prior to antigen recognition. Antibody genes undergo these DNA rearrangements during B-cell maturation, allowing the production of millions of different antibody molecules. This generates a remarkable diversity of antibodies but also introduces the chance of self-reactivity [34]. This self-reactivity is balanced by multiple mechanisms, including receptor revision, B-cell clonal deletion, and anergy [35]. After encounter with antigen, the B-cells differentiate into memory B-cells and plasma cells that can secrete all heavy chain classes [36]. 

 B-cell activating factor (BAFF), a member of the tumor necrosis factor receptor (TNF-R/TNF) superfamily, is expressed on the B-cell surface, and a related molecule, a proliferation-inducing ligand (APRIL), is also effective regulator of immunity [37]. BAFF appears to have a role in the induction of B-cell maturation, class switch recombination of naïve IgM + IgD + B-cells, and is a costimulator of T cells [37]. APRIL is similarly involved in class switch recombination and seems to have a costimulatory effect on B-cells [38]. Serum BAFF concentrations have been noted to be increased in autoimmune disease [39] Animal studies with BAFF and APRIL inhibitors have shown a reduction in hyperthyroxinemia and TSH receptor antibodies in a murine model of Grave's disease [40]. In humans with Grave's disease, the BAFF levels significantly correlated with Tg-Ab but not with TPO-Ab nor TSHR-Ab [41].

### 3.3. Other Factors Affecting Immunoglobulin Secretion

Murine studies have shown that T cells can induce the secretion of immunoglobulins [42] IL-4 a cytokine produced by Th cells affects class switching and may suppress immunoglobulin secretion [43]. TGF-*β*, secreted primarily by activated T cells, may also have a role in class switch recombination [44].

 CD40 is a costimulatory protein that is found on many antigen presenting cells. The interaction of CD40 with CD40L on the T cell provides essential costimulatory signals to B-cells, allowing immunoglobulin class switching, and germinal cell formation [45]. B7 is another protein found in antigen presenting cells that is important in full activation of T cells, that may have a role in immunoglobulin secretion. When paired with CD28 in T cells, this may affect baseline limmunoglobulin levels and alter the relative proportions of immunoglobulin subclasses [46].

Transcription factors involved in regulation at the level of immunoglobulin-secreting plasma cells include BLIMP1 (B-lymphocyte-induced maturation protein 1) and XBP-1 (X-box binding protein 1). BLIMP1 regulates the secretion through decreased transcription of immunoglobulin heavy chain and light chain genes and also influences the J chain. XBP1 induces gene expression leading to increase in the size of the B-cell, the mitochondria, and the size of the endoplasmic reticulum, as well as having direct actions on the endoplasmic reticulum itself [47]. 

### 3.4. Regulation of Thyroid Autoantibody Secretion

In autoimmune thyroid disease, it appears that Tg-Ab and TPO-Abs are more a response to thyroid inflammation than the actual cause as evidenced by their polyclonality and their failure to induce disease when transferred to animal models. However, it seems that at least in animal studies in both obese chicken [49] and NOD mice [50] thyroid antibodies develop spontaneously with no clear precipitator. It is still unclear what first induces thyroid antibody production in humans, but a few important observations have been made. For example, thyroid antibodies are less common in children. Studies performed in Indian and Greek peripubertal boys and girls [51, 52] have noted TPO-Ab positivity at only ~4.5%, with antibodies most prevalent in postpubertal girls [52]. In the geriatric population, it is known that autoantibodies tend to increase with age [53]. However, one small cross-sectional study comparing female centenerians with younger women noted the reduced occurrence of Tg-Ab and TPO-Ab in the centenerians [54] so it is possible that with extreme age there is a loss of autoantibody production or that the survivors have less evidence of autoimmune responses. 

Few longitudinal studies exist in the population as with regards to conversion to thyroid antibody positivity. Using the original Whickham cohort, a twenty-year followup showed that 21% of women (at the time, 55–65 yrs) had developed antithyroid antibodies, at an age range corresponding to the time of menopause [27] and the same cohort showed that individuals who had been thyroid antibody positive originally were noted to have developed increased titers as they grew older [55].

Environmental factors may induce or suppress the development of autoantibodies (Table 2). The Hygiene Hypothesis has been supported by observations of increased thyroid antibodies in more affluent towns [56] suggesting that wider exposure to infection may reduce autoimmunity. A study in Amsterdam involving euthyroid relatives of AITD patients without antibodies to Tg or TPO noted an increase in seroconversion on discontinuation of smoking [57]. Studies comparing appropriate age and sex matched adolescents 13–15 years after the Chernobyl nuclear accident in 1986 versus areas without exposure have shown a TPO-Ab and Tg-Ab prevalence of 8.5%. Taken individually, TPO-Abs were more prevalent in exposed areas, while Tg-Abs did not show a significant difference. It is interesting to note that combined TPO-Ab and Tg-Ab prevalence was much less [58] compared to studies performed earlier which showed a prevalence of 19.5% (6–8 years after event) [59]. There was a rise after exposure, and progressive attenuation which is similar to the effects noted after radioiodine therapy in Grave's disease, as will be discussed later [58]. Thyroid autoimmunity and radioiodine did not have a dose-response relationship in a study on Hiroshima and Nagasaki Atomic bomb survivors 60 years after the event [60].

Iodine and iodine containing drugs such as amiodarone contribute to increases in thyroid autoantibodies as shown in many studies [61]. In Denmark, comparing two appropriately matched cross-sectional groups with and without iodine supplementation showed a marked increase in thyroid antibodies with iodine supplementation probably secondary to the increased iodination of thyroglobulin which enhances its immunoreactivity [62]. 

Improvement from immune collapse in patients with HIV may induce the appearance of thyroid autoantibodies and AITD and treatment of hepatitis c with interferon alpha has had similar effects likely secondary to a direct influence on the thyroid cell [63].

In patients with Hashimoto's disease, the level of autoantibodies is affected by Selenium intake. Selenium deficiency seems to affect both the rate of thyroid cell necrosis [64] and immune function [65]. In a randomized, prospective blinded study in chronic autoimmune thyroiditis patients, selenium supplementation decreased TPO-Ab but not Tg-Ab [66]. 

In Grave's Disease a randomized prospective study compared TSHR-Ab in medical therapy, surgical therapy, and radioiodine therapy and showed a progressive decrease in titers in both medical and surgical therapy with gradual disappearance in 18 months in 70–80% of the patients. Radioiodine therapy induced a 1-year worsening of autoimmunity, while medical therapy showed a rebound after discontinuation of medications [67, 68]. Tg-Ab and TPO-Ab were also increased after radioiodine treatment [69]. It has been previously theorized that this is partly due to the temporary increase in available autoantigens for the immune system [70], and an alternate theory is the direct effect of radioiodine on the immune system, especially on the suppressor T Cells [69, 71].

### 3.5. Changes in Pregnancy

**Figure 2:**
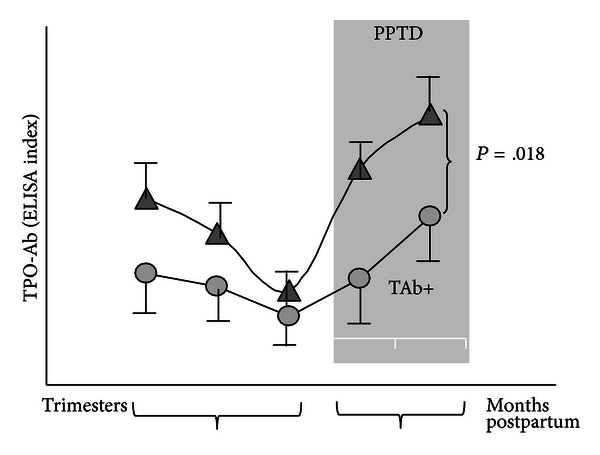
Thyroid antibody levels throughout pregnancy. There is a fall in thyroid autoantibodies reaching its nadir in the third trimester, before rebounding postpartum, in what is clinically seen as postpartum thyroid disease (PPTD: postpartum thyroid disease). Reproduced from [48].

Pregnant patients screened during the first trimester of pregnancy have an increased prevalence of thyroid autoantibodies (up to 20%) reflective of the greater prevalence in this age group rather than secondary to the pregnancy itself [48, 72]. In fact the physiologic changes surrounding pregnancy include changes allowing an immunotolerant environment for the *nonself* fetus, and this results in a decline in autoantibodies. So in pregnancy there is a marked fall in both TPO-Ab and Tg-Ab levels followed by an increase in the postpartum (Figure 2) [48, 73]. It is now clear that many factors may have a role in creating this immunotolerant environment.

#### 3.5.1. The MHC

All autoimmune diseases have shown a genetic association with the HLA gene locus as manifested by the prevalence of certain HLA alleles in the patient populations [74]. For example, Graves' disease has been widely associated with HLA-DR3 in Caucasians and further investigation has suggested that it is residue 74 (Arginine) in the molecule which is the major influence [75]. The fetomaternal interface, which includes the villous trophoblasts and syncytiotrophoblast microparticles, avoid allogeneic responses because they lack HLA class I and class II proteins and only express other MHC molecules such as HLA-S, -E, -F, and HLA-G [76] which are thought to confer resistance to NK cells [77].

#### 3.5.2. T Lymphocytes

Animal studies have clearly demonstrated the important role of T cells in the context of pregnancy and tolerance. They function in an antigen-specific manner to limit maternal immune responses to the fetus [78]. Although studies of CD4+ T cells in human pregnancy have shown very modest changes in absolute numbers [79], the Th1/Th2 ratio has been proposed to indicate a successful pregnancy as a Th2 phenomenon, with the Th2 chemokines downregulating the Th1 response [80]. However, this Th1/Th2 dichotomy was not able to explain the multitude of immune responses in pregnancy among them is the fact that some Th1 cytokines are necessary in some aspects of pregnancy [81] and it did not explain the suppression of autoantibodies, including thyroid autoantibodies. We now know that the CD4+ CD25+ FoxP3+ regulatory T cells are the most potent and widespread lineage of immune cells that are capable of regulating immune function [82]. Treg cells may proliferate peripherally after encountering foreign antigens (such as fetal antigens) and migrate towards the feto-maternal interface generating a tolerant environment characterized by cytokines such as TGF-*β*, Leukemia Inhibitory Factor (LIF), and HO1 which are proposed to protect pregnancy [83]. Hence, increases in Treg cells during pregnancy have been well documented and clearly are major contributors to the dampening of autoimmune responses in pregnancy. It is also likely that deficiency in their function or proliferative responsiveness may endanger a pregnancy [84].

#### 3.5.3. B-Cells

B-cells are not only capable of antibody secretion but are also potent antigen presenting cells interacting with the T-cell receptors [86]. However, there appear to be no significant changes in the quantity of B cells in the circulation during normal human pregnancy yet one unifying characteristic is the fall in autoantibody titers (Figure 2) most likely secondary to the increase in Treg cells. However, estrogen has also been shown to deliver a negative signal towards B-cell function in pregnancy [87], and this effect is lost in the postpartum. Newly described B-regulatory cells discussed above have also been shown to modulate the immune response and inflammation [88] but their role in pregnancy still remains to be elucidated. 

## 4. Influence of Thyroid Antibodies on Reproduction

### 4.1. Fertility and Miscarriage

**Figure 3:**
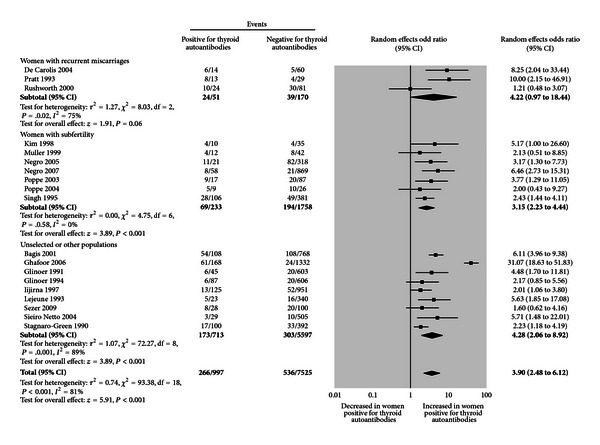
Relationship between thyroid antibodies and Miscarriages. The association of thyroid autoantibodies and miscarriages in multiple cohort studies is shown in meta-analysis by Thangaratinam et al. 2011 [85]. There is an increased prevalence of women positive for thyroid autoantibodies in individuals with subfertility and with recurrent miscarriages.

The absolute inability to conceive after approximately 1 year of regular intercourse without contraception is defined subjectively as infertility [89]. A pooled review of the multiple studies involving autoimmune thyroid disease (AITD) in women with infertility compared to controls showed an overall relative risk of infertility of 2.1 (*P* < 0.0001). However, this should be interpreted cautiously as these studies were retrospective reviews with different causes of infertility and different assays for detecting thyroid antibodies [90]. Infertility may be due to the hormonal changes associated with AITD or to problems with implantation of the embryo [91], reflected more clearly in established pregnancies by early miscarriages. The studies of Stagnaro-Green [48] in New York in 1990 and confirmed by Glinoer et al. [92] in Belgium and in multiple subsequent studies have demonstrated increases in the miscarriage rate in women who are thyroid autoantibody positive and such women are also more likely to deliver early [93]. A meta-analysis ascertaining this relationship showed an odds ratio (OR) of 2.73 (95% CI 2.20–3.40) in 8 case controls and 10-longitudinal studies (Figure 3) [94].

As mentioned earlier, a number of etiologies have been hypothesized as the cause of the relationship between spontaneous termination of pregnancy and autoimmune thyroid antibodies. These include (1) the existence of a subtle degree of hypothyroidism, (2) thyroid antibodies reflecting an autoimmune imbalance in the pregnant female, (3) thyroid autoantibodies acting directly on the placenta or the fertilized ovum causing rejection, or (4) women with thyroid autoantibodies becoming pregnant at an older age, hence with an increased risk. Mild thyroid failure has been implicated in thyroid antibody positive women who have miscarriages and are euthyroid as judged by routine thyroid function tests, since higher levels of TSH within the normal range have been noted in meta-analysis studies [94]. One study showed a marked reduction in miscarriages when thyroid antibody women were treated with thyroxine [95]. 

### 4.2. Endometriosis

Of interest, AITD may also occur in patients with endometriosis which has a frequent association with infertility [96]. In the study of Poppe et al., involving 197 women with a female cause of infertility, they found 11% were attributed to endometriosis. Of these 11%, 29% were TPO Ab+, which was a significantly higher prevalence compared to controls [97]. Endometriosis has also been associated with immunological changes, including possible endometrial autoantibodies [96] and deposition of complement, cytotoxic effects on the endometrium, and declining levels or functional defects in natural killer (NK) cells [98]. Such NK cell dysregulation may activate Graves' disease or Hashimoto's thyroiditis [99]. 

### 4.3. Influence of Thyroid Antibodies on IVF Failure

It was Geva et al. that first described the possible correlation of *in vitro fertilization (IVF)* and thyroid antibodies in a study examining organ-specific autoimmune antibodies arguing for its possible role in predicting poor success for IVF, in such patients [100]. The pregnancy rate in women with thyroid antibodies, in their study, was only 13.6%. In a prospective cohort study, 234 women were screened for TPO-Ab, serum TSH, and Free T4 prior to the first cycle of IVF, and there was a 50% miscarriage rate in females with thyroid antibody positivity but only a 23% rate of loss in those who did not [101]. The hypotheses that have been suggested for this relationship are similar to those of miscarriage in normal pregnancy discussed earlier. However, one study of the use of levothyroxine in IVF in euthyroid women did not show any differences in the frequency of miscarriages compared to untreated controls suggesting a different pathophysiology [102], but a recent meta-analysis of 220 patients in 3 randomized control trials showed improvement in the rate of delivery and embryo implantation in LT4 supplemented patients versus placebo treated patients and a decreased rate of miscarriage [103].

## 5. Thyroid Failure and Thyroid Autoantibodies

### 5.1. Definitions

Hashimoto's thyroiditis is often defined as the simple presence of serum thyroid autoantibodies which, pathologically, correlate well with an intrathyroidal lymphocytic infiltrate [104]. It requires more than 75% of thyroid function to be compromised before thyroid failure is reflected in changes in thyroid function testing and so newly diagnosed patients with thyroid failure often have had the disease for many years. Hence, efforts to design studies to discover precipitating events have been difficult in gathering a population to study because of the time lag between the development of actual thyroid pathology and biochemical or clinical symptomatology. 

### 5.2. Prevalence in Pregnancy

Hypothyroidism, defined by an increase in TSH, is found in 4.6–5% of all pregnancies [105]. Hashimoto's thyroiditis, even with the varying definitions, is the most common cause of such hypothyroidism in pregnancy [106, 107]. Hence, there are a large number of children who have been exposed to thyroid autoantibodies in utero and who may have been hypothyroid during development [108, 109].

### 5.3. Outcomes in Maternal Hypothyroxinemia

It was Man (1972) who initially described how maternal hypothyroxinemia may have profound effects on the intellect of the children without the presence of classic cretinism [111]. Since then, multiple studies have supported the association between overt maternal hypothyroidism and abnormal development of the neural system in the offspring. A large retrospective study involving ~25,000 pregnant women and their children tested at ~8 years of age, using multiple developmental tests including IQ, showed that the children of untreated hypothyroid mothers had IQ levels more than 1 standard deviation below those children born to control mothers [108]. More recent studies have confirmed this effect—with intellectual and motor development differing even when measured as soon as 25–30 months [112]. The evaluation of offspring from more mildly affected mothers who were not diagnosed with Hashimoto's thyroiditis preconception and went through pregnancy without treatment has showed similar intellectual disparities as seen in the study by Haddow et al. [108]. In a prospective, three-year, study by Pop et al. children of mothers with low Ffree T4, but TSH within their normal range, had a delay in the development of mental and motor skills at ages of 1 and 2 years. In the same study, children whose mothers had their T4 increased in the latter part of pregnancy did not show any delay in their child's development [113]. However, a recent interventional trial of mildly affected women, where the mean TSH of affected mothers was only 3.5 uU/mL, failed to show any difference in developmental testing and IQ between the children of treated and control mothers at 3.5 years of age [105]. To date, therefore, the timing, level, and duration of thyroid failure needed to affect intellectual development in the long-term remain unclear. 

### 5.4. Perinatal and Obstetric Outcomes with Thyroid Antibodies

The possible effect of TPO antibodies in perinatal and obstetric outcomes was studied in ~17,000 euthyroid thyroid peroxidase antibody positive and negative women which revealed a fourfold increase in the incidence of placental abruption [114]. A meta-analysis of 11 studies showed that the overall combined relative risk of preterm delivery for pregnant women with TPO-Ab was also significantly increased (RR = 1.98 95% CI 1.29–3.04 *P* = 0.002) (excluding women with any thyroid dysfunction) [115]. 

### 5.5. Thyroid Antibodies and Cognitive Development

TPO and Tg antibodies are noted to be prevalent in up to 20% of the pregnant population and cross the placenta [107]. Despite the wide interest in the concept of Hashimoto's encephalitis [116], there have been few studies examining the influence of thyroid autoantibodies themselves and cognitive development in the offspring. While Haddow's study did not show any correlation between cognitive outcomes and maternal TPO antibodies [108], another study has suggested differences in the offspring with regards to perceptual performance on general, cognitive, and motor scales [109]. It is unclear whether TPO antibodies are just markers for thyroid function, rather than having an effect per se' on neonatal development, but to date there is no biological evidence to suggest a mechanism for an influence of thyroid autoantibodies to Tg and TPO on the intellectual development of the fetus.

### 5.6. Postpartum Autoimmune Thyroiditis

The ATA Guidelines have defined postpartum thyroiditis (PPT) as the occurrence of thyroid dysfunction in the first postpartum year in women who were euthyroid prior to pregnancy [118]. postpartum thyroid dysfunction is a more encompassing term which defines thyroid dysfunction ranging from postpartum thyroiditis to postpartum Grave's Disease. In those women who are TPO antibody positive, there is a noticeable rise in levels postpartum [119]. In addition, thyroid autoantibodies serve as the principal clinical predictor of the development of postpartum thyroiditis (PPT) in the 4–12 months postpartum (Table 3) [117]. The measurement of thyroglobulin antibodies has proven to be clinically less rewarding in postpartum thyroiditis. 

This form of thyroid dysfunction resembles Hashimoto's thyroiditis clinically and biochemically except for the fact that it tends to be transient—usually lasting just a few months and passing from a destructive phase with a suppressed TSH level to a hypothyroid phase with an increased TSH level before normal thyroid function returns. A meta-analysis of 15 studies of PPT showed the prevalence rate to be 9.0–11.7%, with higher prevalence in high risk groups such as women with type 1 diabetes mellitus, a family history of thyroid disease, or a prior history of thyroid disease. There may also be geographical differences secondary to genetic or environmental modulation [120]. While 50% of TPO-Ab positive women will develop PPTD, it can also spontaneously arise in the absence of TPO antibodies during pregnancy [121]. Hence, the use of TPO-Ab in screening populations may have utility. In a study of 410 patients. TPO-Ab screening in early pregnancy had a sensitivity of 100%, with a specificity of 41% and a positive predictive value of 48%. This means that almost half of the individuals who screened positive for TPOAb eventually developed postpartum thyroiditis [117].

PPT may become permanent in the post partum or portend thyroid failure in later years [117]. A large prospective study from Southern Italy, an area of mild deficiency, showed an incidence of PPT to be 3.9%, with 50% remaining hypothyroid after 1 year [122]. Smaller studies with a mean followup of around 3–12 years have reported varying levels of permanent hypothyroidism, ranging from 15–46%. In a longitudinal study for 12 years of 71 patients with PPTD, 27 or 38% of the women developed permanent hypothyroidism [123].

## 6. Autoantibodies to the TSH Receptor

**Figure 4:**
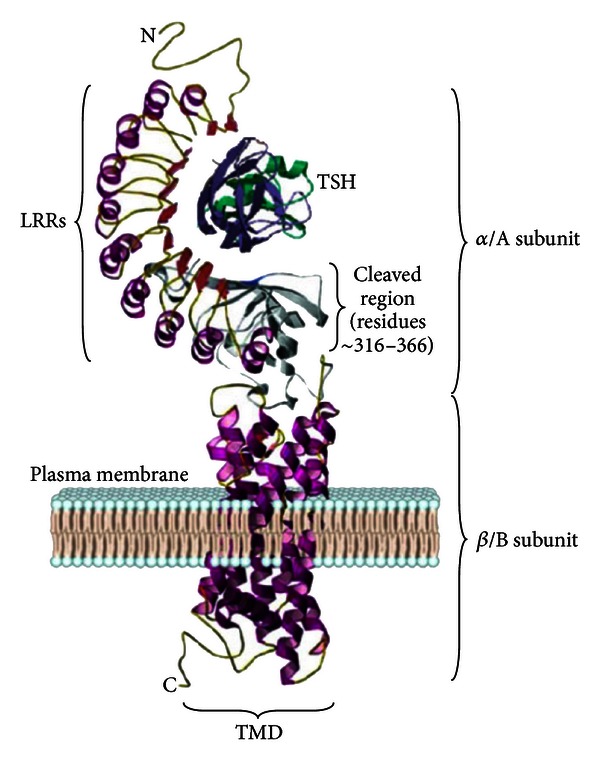
TSH Receptor Structure. The TSHR includes a large extracellular domain (*α* or A subunit) that includes nine leucine-rich repeat (LRR) domains and transmembrane/intracellular domain (b-or B subunit). Adapted from [110].

The thyrotropin receptor (TSHR) is one of the primary antigens in autoimmune thyroid disease, being targeted by antigen-specific T cells and autoantibodies [125]. The TSH receptor is a seven transmembrane G-protein-coupled glycoprotein. It is the largest of the glycoprotein hormone receptors and is similar in structure to the luteinizing hormone and follicle stimulating hormone receptors (Figure 4). [126]. The TSHR consists of a large ectodomain made up of leucine-rich repeat sequences, which is the principal antigenic region, and a transmembrane domain and a linking structure called the hinge region [124]. The TSHR signals via all of the G-protein pathways but induces primarily both the PKA-cAMP and PKC-phospholipase C (Phosphoinositide 3-kinase-AKT-PKC) transduction systems [127].

## 7. Discovery of TSH Receptor Autoantibodies (TSHR-Abs)

TSHR-Abs were initially described by Adams and Purvis in the 1950s [128] as a long acting thyroid stimulator (LATS) in the sera of patients with severe Grave's disease. They were revealed by differences between the long half life of LATS in guinea pigs compared to the shorter half life of TSH and their work laid the groundwork for our understanding of Grave's disease today [129, 130]. However, it took two decades until Smith and Hall provided the direct evidence that this stimulating IgG was in fact due to the existence of TSH receptor autoantibodies [131]. And it took another three decades to develop a human monoclonal thyroid stimulating autoantibody derived from lymphocytes of a patient with Grave's disease [132]. 

## 8. The Three Types of TSHR Antibodies

**Figure 5:**
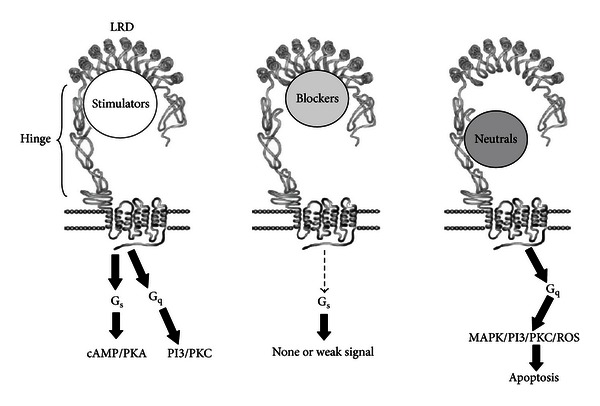
Types of TSH receptor antibodies. Three types of TSH receptor antibodies with their corresponding signal cascades. Adapted from [124].

Since TSHR signal propagation is classically mediated through the G-protein effectors, G*α*s and G*α*q, these signaling pathways have been used to investigate TSHR-Abs in patients with AITD [133]. Using the generation of intracellular cAMP as the end point, three types of TSHR antibodies have been characterized (Figure 5). Stimulating TSHR-Abs are the classical LATS antibodies which increase cAMP levels via the TSHR. Blocking TSHR-Abs reduce or totally block the action of TSH increasing cAMP generation and may also be found in patients with Hashimoto's thyroiditis [134], and “neutral” TSHR-Abs which do not influence TSH action have no effect on TSH binding nor cAMP levels but which signal through the PKC pathway and can induce cell apoptosis [135]. TSHR-Abs have a number of common characteristics. The stimulating variety is mostly restricted to IgG subclass 1; they are present in low titers with specific B-cells in low frequency, but the antibody affinity tends to be high [136].

## 9. Measuring TSHR-Abs

In the absence of Graves' orbitopathy, the diagnosis of Graves' disease can be made by hyperthyroid function tests in the presence of TSHRAb. Alternatively, the diagnosis can be indirectly concluded with hyerthyroidi function tests in the presence of a diffuse thyroid gland (by sonography or scanning) and a normal or increased 24 hour radioiodine uptake. TSHR-Ab testing adds considerably to the clinical picture since it is helpful in monitoring the response to treatment and in predicting the course of the disease [137]. In pregnancy, as we will discuss further, it is especially helpful in predicting the risk of neonatal thyroid dysfunction [138].

 Currently, the assays available to detect TSHR-Abs in patients with Grave's disease reach a sensitivity of ~80% but with a specificity of 100% [139] TSHR-Abs are not found in any disease other than AITD. There are two clinically available methods for measuring TSHR-Abs. The simplest and most precise approach is the receptor assays. These measure the inhibition of labeled TSH or a labeled monoclonal TSHR-Ab binding to solubilized TSHRs. While such assays can be automated and are cheap to perform, they do not differentiate between stimulating or blocking TSHR-Abs. Of course, in clinical practice this is not an issue, since the patient is the bioassay. If the patient is hyperthyroid we do not need a laboratory test to tell us that the TSHR-Ab is primarily of the stimulating variety.

 In pregnancy, when we are concerned with the thyroid status of the neonate, it can be helpful to know the biological action of any persistent TSHR-Abs and so biological assays are available commercially. These measure the stimulation or the inhibition of TSHR activity by measuring cAMP as the end point [140]. These assays are sensitive but are expensive and imprecise. The information gathered is clinically useful since either stimulation or inhibition of TSHR activity has different effects on the neonate.

## 10. Stimulating TSHR-Abs and Pregnancy

TSHR-Abs cross the placenta and can induce neonatal thyrotoxicosis. However, pregnancy usually suppresses Grave's disease. Although exacerbations early in pregnancy are sometimes observed [141] once the first trimester is passed the immune suppression of pregnancy sets in, the Treg cells increase their effectiveness, and the disease very often abates. The aggravation of thyrotoxicosis in the first part of pregnancy is probably secondary to the added effects of hCG which itself can cause gestational thyrotoxicosis [141–143]. In keeping with this scenario, TSHR-Abs reach their lowest point at the time of delivery [144, 145]. Hence, the dose of antithyroid drugs is rapidly decreased during pregnancy so that by the 3rd trimester a third of patients are taking none at all [145, 146]. This means that women have to be monitored for their TSHR-Ab status until they are negative.

Since many patients with Graves' disease have stimulating, blocking and neutral TSHR-Abs, their interplay during the immune suppression of pregnancy can be extremely complex. For example, blocking antibodies have been noted to increase during pregnancy suggesting this as an explanation for the consequent clinical remission of thyrotoxicosis [147]. However, as pregnancy suppresses humoral immunity, all types of TSHR-Ab should be depressed. While many studies have confirmed this, there remain contrary findings which may depend on a woman's individual immune response to the pregnancy [147–149].

## 11. Postpartum Recurrence of Graves' Disease

Titers of TSHR-Abs may increase in the post partum after the loss of the immune suppression of pregnancy. This tends to be seen most commonly from 4–12 months post partum and may lead to recurrence, or even the new onset, of Graves' disease. This occurs at the same time as women may alternatively develop post partum thyroiditis, which may also depress the serum TSH early in its pathology [119]. Hence, women with a suppressed TSH and all those with a history of Graves' disease need to be screened carefully in the post partum for the presence of TSHR-Abs [150]. There has been no correlation between the levels of TSHR-Abs before, or early, in pregnancy and the initiation of postpartum Grave's disease, once again indicating the importance of the women's own immune response to their pregnancy [151]. 

## 12. Perinatal/Fetal Repercussions of TSHR-Abs

Fetal hyperthyroidism is suspected in fetuses with heart rates >160 and intrauterine growth retardation [152]. This requires the presence of both maternal IgG in sufficient quantities and a thyroid that is mature enough to respond [153]. The fetal thyroid gland matures after about 7 weeks although functionally its capacity to concentrate iodide and synthesize T4 does not occur until around the 11th week [154]. And fetal hyperthyroidism does not clinically manifest until the latter part of pregnancy [145]. The ATA has recommended measurement of TSHR-Ab by 24–28 weeks for detection of high risk pregnancies, with three times the upper normal value as an indication for closer followup for the fetus. Serial ultrasounds are recommended, while cordocentesis has been used in very rare situations where the thyroid status of the fetus was unclear [118]. 

## 13. Neonatal Repercussions of TSHR-Abs

Transient neonatal hyperthyroidism caused by transplacental passage occurs rarely in the context of maternal Grave's disease and develops <1% of the cases. Since the half life of IgG is ~3 weeks, then the neonatal dysfunction slowly recovers after this time. It is suspected in neonates presenting with tachycardia, hyperexcitability, and poor weight gain. [155]. Transplacental passage of TSHR-Abs [156] has been titrated to the development of neonatal hyperthyroidism to such a careful extent that the maternal level can be used as a predictor of neonatal dysfunction [157]. This is clinically important in those women whose maternal levels of TSHR-Abs fail to diminish in the third trimester [145]. 

The transplacental passage of blocking TSHR-Abs [145] may, by contrast, cause *transient hypothyroidism* [158] in the neonate. There are cases of these transplacentally transferred antibodies causing delayed development of the infant's thyroid gland with a slow recovery of normal thyroid function in the post partum [159]. It has been estimated that ~2% of congenital hypothyroidism may be caused by TSH receptor blocking antibodies [160]. 

## 14. Thyroid Autoantibody Screening in Pregnancy

The detection of thyroid antibodies before or early in pregnancy can predict the development of pregnancy loss, the need for thyroxine replacement therapy during pregnancy, and the onset of post partum thyroiditis [117]. Yet the latest American Thyroid Association guidelines [118] only comment on the lack of evidence needed to make any recommendations for the screening of thyroid autoantibodies in pregnancy. In addition, the Endocrine Society [161] still does not recommend screening for these antibodies in pregnancy. At least the guidelines do define the utility of measuring thyroid autoantibodies in some situations. For example, in postpartum depression, TPO Ab testing has been advocated. In euthyroid patients who are TSHR-Ab positive, all agree that close monitoring in pregnancy is needed.

## 15. Conclusions

Pregnancy complicates both the physiology of the thyroid cell and the immune response to thyroid antigens. Thyroid autoantibodies are involved in both these areas and are associated with major alterations in the course of pregnancy affecting the mother, the fetus, and the neonate. Thyroid antibodies can serve as useful clinical predictors of thyroid dysfunction, for example, TPO-Ab and post partum thyroiditis and TSHR-Abs and neonatal hyperthyroidism. But why humans are so susceptible to the development of thyroid autoantibodies remains uncertain.

## Figures and Tables

**Table 1:** Tg-Ab and TPO-Ab as clinical markers.

	Tg-Ab and TPO-Ab as clinical Markers
Positivity	More likely to be TPO-Ab than Tg-Ab if serum positive for only one autoantibody

Prevalence	TPO-Ab are more prevalent than Tg-Ab in the community, and in Grave's disease

Titers	Tg-Ab and TPO-Ab fluctuate in parallel, decreasing after antithyroid drugs, increase transiently after I^131^ therapy, decrease during pregnancy, increase post-partum, and are induced/enhanced by interferon-*α*

Predictors	TPO Ab Better than Tg-Ab in predicting post-partum thyroid dysfunction

Disease	Tg-Ab alone not significantly associated with disease

The comparison of thyroglobulin and TPO antibody as used as clinical markers. Adapted from McLachlan and Rapoport 2004 [18].

**Table 2:** Regulation of thyroid antibodies in euthyroid and individuals with active thyroid disease.

	Downregulated	Upregulated
Euthyroid individuals	(i) Extremes of ages (ii) Pregnancy	(i) Smoking discontinuation (ii) Improvement in immune collapse in HIV (iii) Hep C treatment with interferon (iv) Nuclear fallout (v) Iodine supplementation (vi) Amiodarone, and iodine containing drugs

With thyroid disease	(i) Pregnancy (ii) Radioiodine, after 1 year (iii) Antithyroid drugs (iii) Selenium	(i) Postpartum (ii) Postradioiodine treatment

The table summarizes some of the various agents/events that may downregulate or upregulate thyroid autoantibodies (both TPO and Tg-Ab) in both euthyroid individuals and those with active thyroid disease. Abstracted from [35–47, 49–55].

**Table 3:** Sensitivity, specificity, and positive predictive values of thyroid autoantibodies when sampling done at the different times in pregnancy and post-partum adapted from Premawardhana et al. The positive predictive value of thyroid antibodies in predictive post-partum thyroid disease [117].

Investigator	Antibody	Ab-positive subjects (Ab-negative subjects with PPTD)	Sampling time—(pp) postpartum *in months *	Sensitivity	Specificity	PPV
Jansson	Micro	44 (7)	2–5	0.77	0.95	0.52
Amino	Micro	61 (1)	3	0.89	0.92	0.4
Lervang	Micro	38 (3)	3	0.87	0.97	0.53
Vargas	Micro	41*	Delivery	0.45	0.95	0.63
Vargas	Micro	54 (11)	2–4	0.76	0.91	0.65
Vargas	Micro	69 (8)	5–7	0.86	0.9	0.68
Pop	Micro	15 (6)	Trimester 3	0.71	0.92	0.52
Kuijpens	TPO	31 (5)	Trimesters 1/3	0.67	0.93	0.31
Kent	TPO	55 (31)	6	0.64	0.95	0.64
Kent	Micro	40 (46)	6	0.46	0.98	0.78
Sakaihara	Micro/Tg	50 (65)	Early pp-1	0.14	0.88	0.07
			Early pp-3	0.37	0.90	0.21
Solomon	Micro	55*	Delivery			0.73
Nohr	TPO	66*	Trimester 1	—	—	0.55
Premawardhana	TPO	308 (0)	Early pregnancy	1.00	0.62	0.48
			Early Postpartum	1.00	0.41	0.48

Sensitivity is defined as the number of true positives as a proportion of the sum of the number of true positives and false negatives. Specificity, defined as the number of true negatives as a proportion of the sum of the false positives and true negatives. Positive predictive value (PPV) is defined as the number of true-positives as a proportion of the sum of true and false positives.

*Unknown or not included.
